# Sex differences in spontaneous reports on adverse drug events for common antihypertensive drugs

**DOI:** 10.1007/s00228-018-2480-y

**Published:** 2018-05-27

**Authors:** Diana M Rydberg, Stefan Mejyr, Desirée Loikas, Karin Schenck-Gustafsson, Mia von Euler, Rickard E Malmström

**Affiliations:** 1 Department of Medicine Solna, Karolinska Institutet, Stockholm, Sweden; 2 Clinical Pharmacology, Drug Evaluation Unit, L7:03, Karolinska University Hospital Solna, 17176 Stockholm, Sweden; 3 Department of Clinical Science and Education, Södersjukhuset, Karolinska Institutet, Stockholm, Sweden; 4 Public Healthcare Services Committee, Stockholm County Council, Stockholm, Sweden

**Keywords:** Antihypertensive treatment, Women, Men, Adverse drug events, Sex-differences

## Abstract

**Purpose:**

To explore sex differences in spontaneously reported adverse drug events (ADEs) for antihypertensives in routine care.

**Methods:**

A cross sectional analysis combining number of reports from the national pharmacovigilance database with data from the Swedish Prescribed Drug Register, from 2005 to 2012 for ACE inhibitors (ACE-I) and angiotensin receptor blockers (ARB), with or without thiazide, diuretics (thiazides, potassium-sparing agents, sulfonamides, aldosterone antagonists), selective betablockers, and dihydropyridine calcium-channel-blockers (DHPs). The total number of reports was adjusted to exposed patients and dispensed DDDs among women and men. Dose exposures, co-medications, and co-prescriptions were also analyzed.

**Results:**

In women, a higher prevalence of ADE-reports was seen in ACE-I (odds ratio, OR 1.21; 95% CI 1.09–1.35), ACE-I-combinations (OR 1.61; 1.44–1.79), ARB-combinations (OR 2.12; 1.47–3.06), thiazides (OR 1.78; 1.33–2.39), diuretics and potassium-sparing agents (OR 1.62; 1.22–2.17), and DHPs (OR 1.40; 1.17–1.67), with a potential linkage to dose exposure. For aldosterone antagonists, we observed a higher prevalence of ADE reports in men (OR 0.75; 0.59–0.97) but without any sex difference in dose exposure.

**Conclusions:**

This ecological study of reported ADEs showed a higher prevalence of reports in women in six out of ten groups of antihypertensive drugs, and this may potentially be linked to dose exposure. Aldosterone antagonists was the only group with a higher prevalence of ADE-reports in men with a similar dose exposure between women and men.

**Electronic supplementary material:**

The online version of this article (10.1007/s00228-018-2480-y) contains supplementary material, which is available to authorized users.

## Introduction

Studies have shown that women have a 50–70% greater risk of suffering from adverse drug reactions (ADRs) compared to men, and furthermore patients admitted to hospital with an ADR are in 60% of the cases women [1, 2]. There are differences in pharmacokinetics between women and men, making women in general more susceptible to dose-dependent ADRs [3]. Numerous factors influence the bioavailability and distribution of drugs, such as the ratio of lean to fat tissue, circulating plasma volume, and the amount of plasma proteins binding the drug [4]. On average, the body composition in women includes higher percentage of body fat and a lower body mass [3, 5]; consequently, lipid soluble drugs with a longer half-life and water soluble drugs may yield higher exposure in women. Many drugs are metabolized by enzymes of the CYP system. Sex differences have been shown regarding CYP1A2, CYP2D6, CYP2E1, and CYP3A4 [6] but studies on the clinical impact of these differences are scarce [7]. Renal clearance is usually higher in men than in women [3]. Women may respond to cardiovascular medication differently than men [8], and sex differences in pharmacodynamic responses may include both increased and decreased effects as well as adverse effects in women compared to men. It is possible that these differences, at least in part, may relate to exposure. For example, drug-induced Torsade de Pointes ventricular tachycardia, electrolyte abnormalities with diuretics, dry cough with angiotensin-converting enzyme inhibitors (ACE-I) [6], higher incidence of peripheral edema, and better response of amlodipine [9] are more common in women.

Studies on ambulatory medical populations show women generally reporting more symptoms than men [10, 11]. Women generally report more bodily distress and more frequent somatic symptoms than men [12]; this could even lead to differences in the reporting of adverse drug events (ADEs). However, in a regional pharmacovigilance center in France, there was no sex difference in the incidence of reporting of ADRs overall [13]. Furthermore, no sex difference was seen in suspected ADRs to ACE inhibitors and ARBs in spontaneous reports in the Campania region, Italy [14]. Spontaneous reporting of ADEs is an important tool in obtaining better knowledge about sex differences in ADEs, in addition to the information from the clinical trials conducted before the drug has been introduced on the market. Therefore, we conducted a study to explore sex differences regarding reported ADEs from the ten most commonly prescribed antihypertensive medicines in Sweden, using the Swedish pharmacovigilance database SWEDIS and the Swedish Prescribed Drug Register (SPDR).

## Methods

This was a cross sectional study combining data on reported ADEs from SWEDIS and data on dispensed drugs from the SPDR. An ADR may be defined as harm directly caused by the drug at normal doses and during normal use compared to an ADE which has a wider definition, which includes ADRs, overdoses, dose reductions, and discontinuations of drug therapy [15]. The lack of information about the specific reactions in pharmacovigilance databases made us chose the wider definition, ADEs, when referring to the reports. Data on ADEs was extracted from SWEDIS, which was established in 1965 and contained more than 130,000 spontaneous ADE-reports at the end of December 2012. In Sweden, at the time of the study period, physicians, dentists, and nurses were supposed to report serious ADEs; ADEs not mentioned in the Summary of Product Characteristics (SPC); ADEs related to the use of new drugs (≤ 2 years after authorization) except those already labeled as common in the SPC; and ADEs that seem to be increasing in incidence, to any of Sweden’s six regional pharmacovigilance centers. Specially trained nurses and clinical pharmacologists reviewed the ADE-reports and assessed causality between drug and reaction and if the report was serious or not. For the latter, the following criteria were used: a serious ADE was defined as any untoward medical occurrence that in any dose resulted in death, led to hospitalization or prolongation of existing hospitalization, resulted in persistent or significant disability, or was life-threatening. These criteria were introduced in SWEDIS in January 1998, and from October 2006, ADE-reports were also registered as serious if they were assessed as an important medical event [16]. Consumer reporting, that started in 2008 in Sweden, were at the time collected in a separate database and therefore not included in this study.

In SWEDIS, we extracted both the total amount of ADE-reports and the amount of serious ADE-reports for ten selected groups of antihypertensives; ACE-Is (Anatomical Therapeutic Chemical classification (ATC) [17] code C09AA; e.g., enalapril) and with fixed thiazide combinations (C09BA; e.g., enalapril/hydrochlorothiazide), angiotensin II receptor blockers (ARBs) (C09CA; e.g.losartan), and with fixed thiazide combinations (C09DA; e.g., losartan/hydrochlorothiazide), thiazides (C03AA; e.g., hydrochlorothiazide), low-ceiling diuretics and potassium-sparing agents (C03EA; e.g., amiloride/hydrochlorothiazide), sulfonamides (C03CA; e.g., furosemide), aldosterone antagonists (C03DA; e.g., spironolactone), dihydropyridine derivatives (DHPs) (C08CA; e.g., amlodipine), and selective beta blocking agents (C07AB; e.g. metoprolol). The number of ADE-reports for women and men respectively, were adjusted for dispensed drug prescriptions, using data from the SPDR [18]. This register was established in 1999 and is held by the National Board of Health and Welfare. The register covers all prescribed drugs dispensed to the Swedish population (9.1–9.6 million inhabitants from 2005 through 2012). The selected subgroups of antihypertensives were the ten most commonly prescribed antihypertensive drugs, identified through this register. Since July 1, 2005, all data is registered at an individual level with unique identifiers for all patients. We analyzed data between July 1, 2005 and December 31, 2012. For the time period mentioned above, within the selected groups of antihypertensives, both individuals exposed (the number of individuals with at least one dispensed prescription), and defined daily doses (DDDs), for women and men separately, were obtained. When adjusting for dispensed prescription data, the total number of ADE-reports was used in the primary analysis, with the results for sex differences presented as an odds ratio (OR) (Tables 1, 2, 3, 4, 5, 6, 7, 8, 9 and 10). In this case, we chose to calculate the OR because of the ecological study design. In a former study with the same type of data and design, the ORs were in line with the relative risk [19]. We also did a separate secondary analysis of the reports classified as “serious”, as previously described [19].

**Table 1:** Reports and exposure data for ACE-Is (ATC code C09AA) 2005–2012

	Individuals exposed	Number of million DDDs	Total reports	OR adjusted for individ. exposed^a^ (CI^b^)	OR adjusted for nr of DDDs^a^ (CI^b^)	Serious reports	OR adjusted for individ. exposed^a^ (CI^b^)	OR adjusted for nr of DDDs^a^ (CI^b^)
Total	1,144,397	1899	1291	1.21 (1.09–1.35)	1.61 (1.44–1.79)	671	1.10 (0.94–1.28)	1.45 (1.25–1.69)
Women	534,883	757	666	NA	NA	329	NA	NA
Men	609,514	1142	625	NA	NA	342	NA	NA

**Table 2:** Reports and exposure data for ACE-I/thiazide combinations (ATC code C09BA) 2005–2012

	Individuals exposed	Number of million DDDs	Total reports	OR adjusted for individ. exposed^a^ (CI^b^)	OR adjusted for nr of DDDs^a^ (CI^b^)	Serious reports	OR adjusted for individ. exposed^a^ (CI^b^)	OR adjusted for nr of DDDs^a^ (CI^b^)
Total	156,773	171	101	1.67 (1.13–2.48)	1.76 (1.19–2.60)	52	1.63 (0.94–2.82)	1.71 (0.99–2.96)
Women	68,396	73	57	NA	NA	29	NA	NA
Men	88,377	98	44	NA	NA	23	NA	NA

^a^OR women vs men

^b^0.95 Confidence Interval

**Table 3:** Reports and exposure data for ARBs (ATC code C09CA) 2005–2012

	Individuals exposed	Number of million DDDs	Total reports	OR adjusted for individ. exposed^a^ (CI^b^)	OR adjusted for nr of DDDs^a^ (CI^b^)	Serious reports	OR adjusted for individ. exposed^a^ (CI^b^)	OR adjusted for nr of DDDs^a^ (CI^b^)
Total	599,725	958	447	1.18 (0.98–1.42)	1.24 (1.03–1.50)	189	1.11 (0.83–1.47)	1.17 (0.88–1.56)
Women	308,433	480	248	NA	NA	102	NA	NA
Men	291,292	478	199	NA	NA	87	NA	NA

**Table 4:** Reports and exposure data for ARB/thiazide combinations (ATC code C09DA) 2005–2012

	Individuals exposed	Number of million DDDs	Total reports	OR adjusted for individ. exposed^a^ (CI^b^)	OR adjusted for nr of DDDs^a^ (CI^b^)	Serious reports	OR adjusted for individ. exposed^a^ (CI^b^)	OR adjusted for nr of DDDs^a^ (CI^b^)
Total	240,542	327	130	2.12 (1.47–3.06)	2.18 (1.51–3.15)	60	2.02 (1.18–3.46)	2.08 (1.22–3.56)
Women	119,653	160	88	NA	NA	40	NA	NA
Men	120,889	167	42	NA	NA	20	NA	NA

^a^OR women vs men

^b^0.95 Confidence Interval

**Table 5:** Reports and exposure data for thiazides (ATC code C03AA) 2005–2012

	Individuals exposed	Number of million DDDs	Total reports	OR adjusted for individ. exposed^a^ (CI^b^)	OR adjusted for nr of DDDs^a^ (CI^b^)	Serious reports	OR adjusted for individ. exposed^a^ (CI^b^)	OR adjusted for nr of DDDs^a^ (CI^b^)
Total	419,343	564	222	1.78 (1.33–2.39)	1.69 (1.26–2.26)	137	2.69 (1.77–4.07)	2.55 (1.68–3.86)
Women	248,051	341	160	NA	NA	109	NA	NA
Men	171,292	223	62	NA	NA	28	NA	NA

**Table 6:** Reports and exposure data for diuretics and potassium-sparing agents (ATC code C03EA) 2005–2012

	Individuals exposed	Number of million DDDs	Total reports	OR adjusted for individ. exposed^a^ (CI^b^)	OR adjusted for nr of DDDs^a^ (CI^b^)	Serious reports	OR adjusted for individ. exposed^a^ (CI^b^)	OR adjusted for nr of DDDs^a^ (CI^b^)
Total	244,373	307	256	1.62 (1.22–2.17)	1.48 (1.11–1.98)	163	2.20 (1.48–3.28)	2.01 (1.35–2.99)
Women	163,231	211	196	NA	NA	133	NA	NA
Men	81,142	96	60	NA	NA	30	NA	NA

**Table 7:** Reports and exposure data for sulfonamides (ATC code C03CA) 2005–2012

	Individuals exposed	Number of million DDDs	Total reports	OR adjusted for individ. exposed^a^ (CI^b^)	OR adjusted for nr of DDDs^a^ (CI^b^)	Serious reports	OR adjusted for individ. exposed^a^ (CI^b^)	OR adjusted for nr of DDDs^a^ (CI^b^)
Total	857,345	1086	307	0.96 (0.77–1.21)	1.11 (0.88–1.39)	211	1.03 (0.78–1.35)	1.18 (0.90–1.56)
Women	501,956	598	177	NA	NA	125	NA	NA
Men	355,389	488	130	NA	NA	86	NA	NA

**Table 8:** Reports and exposure data for aldosterone antagonists (ATC code C03DA) 2005–2012

	Individuals exposed	Number of million DDDs	Total reports	OR adjusted for individ. exposed^a^ (CI^b^)	OR adjusted for nr of DDDs^a^ (CI^b^)	Serious reports	OR adjusted for individ. exposed^a^ (CI^b^)	OR adjusted for nr of DDDs^a^ (CI^b^)
Total	258,422	104	246	0.75 (0.59–0.97)	0.60 (0.46–0.77)	178	0.78 (0.58–1.05)	0.62 (0.46–0.83)
Women	149,418	66	125	NA	NA	92	NA	NA
Men	109,004	38	121	NA	NA	86	NA	NA

^a^OR women vs men

^b^0.95 confidence interval

**Table 9:** Reports and exposure data for dihydropyridines (ATC code C08CA) 2005–2012

	Individuals exposed	Number of million DDDs	Total reports	OR adjusted for individ. exposed^a^ (CI^b^)	OR adjusted for nr of DDDs^a^ (CI^b^)	Serious reports	OR adjusted for individ. exposed^a^ (CI^b^)	OR adjusted for nr of DDDs^a^ (CI^b^)
Total	918,184	1419	497	1.40 (1.17–1.67)	1.66 (1.39–1.98)	145	1.34 (0.97–1.87)	1.59 (1.14–2.21)
Women	464,926	658	293	NA	NA	84	NA	NA
Men	453,258	761	204	NA	NA	61	NA	NA

^a^OR women vs men

^b^0.95 confidence interval

**Table 10:** Reports and exposure data for selective beta blockers (ATC code C07AB) 2005–2012

	Individuals exposed	Number of million DDDs	Total reports	OR adjusted for individ. exposed^a^ (CI^b^)	OR adjusted for nr of DDDs^a^ (CI^b^)	Serious reports	OR adjusted for individ. exposed^a^ (CI^b^)	OR adjusted for nr of DDDs^a^ (CI^b^)
Total	1,392,619	1186	871	0.92 (0.81–1.05)	0.94 (0.82–1.07)	501	0.85 (0.71–1.01)	0.87 (0.73–1.03)
Women	726,291	612	436	NA	NA	241	NA	NA
Men	666,328	574	435	NA	NA	260	NA	NA

^a^OR women vs men

^b^0.95 confidence interval

For the different groups of antihypertensives, the most frequently reported ADEs were collected, and the total number of the most frequently reported ADEs were presented in a descriptive manner for women and men respectively, without further statistical analyses (suppl. Tables 1–5). The same calculations were made in serious reports for the collected ADEs above. In addition, all reports were also analyzed by age (0–49 years, 50–74 years, ≥ 75 years). The most frequently reported co-medications for the selected groups of antihypertensives for women and men, respectively, were collected and analyzed. Co-medication was defined being a medication assessed as a “suspected” drug and classified as such in the ADE-report (suppl. Table 6–10). In the SPDR, concomitant prescriptions of other drugs within the selected groups were collected and assessed in two different ways: (a) through analyzing prescriptions dispensed the same day or (b) through analyzing prescriptions dispensed within a 6-month period. We describe the most frequently dispensed co-prescribed antihypertensive drugs within the same day, for our selected subgroups (suppl. Table 11–15). In an attempt to estimate prescribed doses of antihypertensives, both DDDs per dispensed prescription per year and DDDs per individuals exposed per year were calculated for women and men, respectively, divided by age group (0–49 years, 50–74 years, ≥ 75 years).

The study was approved by the Regional Ethical Review Board in Stockholm, Sweden, Dnr 2010/788–31/5.

### Statistics

Descriptive statistics were used, and data are presented as proportions with 95% confidence intervals (CI), where appropriate. Sex differences are presented as odds ratios (OR, women/men) with 95% CI. All statistical analyzes were performed using 2 × 2 contingency tables for calculations of odds ratios on Vassarstats.net (http://vassarstats.net/odds2x2.html).

## Results

In women, a higher prevalence of ADE-reports was seen in six of the ten groups of antihypertensive drugs: ACE-Is, ACE-I with thiazide combinations (Tables 1 and 2), ARB with thiazide combinations (Table 4), thiazides, diuretics, and potassium-sparing agents (Tables 5 and 6) and DHPs (Table 9). For aldosterone antagonists, we observed a higher prevalence of ADE-reports in men (Table 8). For ARBs, sulfonamides and selective beta blockers, there were no statistically significant differences between women and men in the prevalence of ADE-reports (Tables 3, 7, and 10). The ORs for the serious reports were in line with the total reports, for thiazides and diuretics and potassium-sparing agents, with even more accentuated ORs for the serious reports (Tables 5 and 6). The ORs adjusted for the number of DDDs were in line with the ORs adjusted for individuals exposed, in all the subgroups. For ACE-Is, aldosterone antagonists, and DHPs, the ORs for the serious reports adjusted for the number of DDDs showed significant results while the ORs for the serious reports adjusted for individuals exposed did not (Tables 1, 8, and 9).

Hyponatremia was one of the most frequently reported ADEs found in several subgroups involving diuretics, such as ACE-I and ARB with fixed thiazide combinations, thiazides, diuretics and potassium-sparing agents, sulfonamides, and aldosterone antagonists (suppl. Tables 1b, 2b, 3a-d). For ACE-Is, cough was a commonly reported ADE (suppl. Table 1a), and leg edema was the most frequently reported ADE for dihydropyridines(suppl. Table 4) while hyperkalemia was the most frequently reported ADE for aldosterone antagonists(suppl. Table 3d). It can be noted that there were only five and seven reports on gynecomastia for spironolactone and eplerenone, respectively, during the study period.

Most ADEs were reported within the age group of 50–74 years (ACE-Is and ARBs with or without thiazide combinations and DHPs) and ≥ 75 years (thiazides, diuretics and potassium-sparing agents, sulfonamides, aldosterone antagonists, and selective beta blockers). In the age group of 75 years or older, there were more reports from women but when adjusted for prescription data, the prevalence of ADEs were in line with the main findings for each subgroup (data not shown). In general, concomitant dispensed prescriptions of other antihypertensives was more frequent in men when analyzing the different subgroups (suppl. Tables 11–15). E.g., for aldosterone antagonists, the prevalence of co-prescription of ACE-Is or ARBs was higher in men (suppl. Table 13d). In the ADE-reports for ARBs and diuretics with potassium-sparing agents, sulfonamides were co-reported medications in a greater extent in men (suppl. Tables 7a, 8b). For aldosterone antagonists, sulfonamides were co-reported to a higher extent in women (suppl. Table 8d).

In the attempt to estimate dose exposure in women and men, both DDDs per dispensed prescriptions and DDDs per individuals exposed per year were analyzed (data not shown). Overall, doses were higher in men, and the dose exposure analyzed by DDDs/Rxs ranged from 1.07 (lowest difference between men and women) to 1.43 (highest difference between men and women). For thiazides, ACE-I/thiazide combinations, ARB/thiazide combinations, and diuretics with potassium-sparing agents, the difference in exposure was lower between women and men (1.07–1.12; DDDs/Rxs for m/w), and this coincided with a larger difference in the prevalence of ADE-reports (more frequent in women) between the sexes. For ACE-Is, sulfonamides, DHPs, selective beta blockers, and ARBs, the difference in exposure was higher between women and men (1.23–1.43; DDDs/Rxs for m/w), and this in turn coincided with smaller differences in the prevalence of ADE-reports between women and men. For aldosterone antagonists, the sex difference in dose exposure was small (1.08), and men had a higher prevalence of ADE-reports in both age groups with enough reports to allow subgroup analyses (50–74 and ≥ 75 years).

## Discussion

The results from our study show a higher prevalence of ADE-reports for women in six of the ten subgroups of common antihypertensives. Only aldosterone antagonists had a higher prevalence of reported ADEs in men. Our findings are in line with the greater risk of ADR-related hospital admissions found in women [1, 2, 20], although data on sex and/or gender differences in the spontaneous reporting of ADEs to pharmacovigilance centers are somewhat more sparse and point to women generally reporting more symptoms than men [10, 11]. From a mechanistic standpoint, our findings are plausible given the differences between men and women in pharmacokinetics and exposure, with women frequently at higher risk for dose-dependent ADRs [4].

The higher prevalence for reported ADEs in women for both ACE-I, ACE-I with combinations, and ARB with combinations, found in our study, was not in line with results from the results from a regional pharmacovigilance center in Italy, where no substantial sex differences were found with regard to suspected ADRs to ACE-Is and ARBs in spontaneous reports [14]. In the Italian study, the fact that ACE-Is and ARBs were prescribed to a greater extent in men during the study period was discussed [14], and the lack of adjustment to prescription data may explain why their results differ from ours. The results from our study are in line with the literature referring to pharmacokinetic and pharmacodynamic differences, giving relatively higher exposure in women to a given dose [3]. These differences, only partly due to sex hormones, may lead to women being more susceptible to ADEs [6]. In the case of ACE-I and ARB, the different effect of sex hormones on the renin angiotensin aldosterone system (RAAS), could also be part of the explanation. Sex hormones, such as exogenous and endogenous estrogens and androgens, interact with the RAAS in opposite ways, with estrogens downregulating and androgens upregulating RAAS [21]. Whether these hormonal influences on the RAS modulate effectiveness and safety of ACE-I or ARBs, have not been established [6]. Despite the higher prevalence of total ADE-reports for ACE-I in women, the number of dispensed prescriptions of ACE-I was in fact lower in women compared to men. Adverse effects of ACE-Is, especially dry cough being more frequent in women compared to men have been found in the literature [22–27], which could partially also explain our findings. The role of ACE in the metabolism of bradykinin has been proposed as a pathogenic mechanism [28], and the effect of genetic polymorphisms in bradykinin receptors and ABO genes, related to ACE levels and associated with ACE-I-related cough, has been found more pronounced in women [29].

Thiazides and diuretics with potassium-sparing agents similarly had a higher prevalence of total ADE-reports in women. This may, at least partially, be explained by women being more susceptible to drug-induced hyponatremia and other electrolyte disturbances due to drug treatment [30]. Female sex is one of the risk factors of thiazide-induced hyponatremia [31], and thiazide-induced hyponatremia is four times more common in women than in men [32]. The mechanisms of drug-related hyponatremia have been postulated to be associated with the syndrome of inappropriate secretion of antidiuretic hormone (SIADH), i.e., hyponatremia caused by increased antidiuretic hormone (ADH) secretion in the presence of normal circulating blood volume. In the case of thiazides- and amiloride-induced hyponatremia and ACE-Is associated to hyponatremia, the proposed mechanism is increased ADH-secretion [33]. The number of individuals exposed to both thiazides and diuretics with potassium-sparing agents was higher in women, which could also contribute to our results.

Although calcium channel blockers, such as amlodipine, are also known to cause hyponatremia [34], this was not among the most frequently reported ADEs in our study cohort. Our findings for DHPs with higher prevalence of ADE-reports in women, are in line with the safety results from a clinical trial with patients with amlodipine, where leg edema was more common in women [9]. In the same study, a greater blood pressure (BP) response to amlodipine was seen in women. Altogether, this indicates that drug exposure is central, translating into efficacy and safety. For DHPs, the number of individuals exposed did not differ between the sexes, ruling out the bias of different prescription patterns contributing to the found sex difference in this case. Aldosterone antagonists were the only group of antihypertensive agents with a higher prevalence of ADE-reports in men. The higher prevalence of co-prescription of aldosterone antagonists together with either ACE-Is or ARBs in men, found in our study, with the risk of hyperkalemia, being part of an explanation. Non-selective mineralocorticoid receptor antagonists (MRAs, e.g., spironolactone), but not selective MRAs (e.g., eplerenone) increased the risk of gynecomastia sevenfolded. Unfortunately, data on women and men were not shown [35]. For aldosterone antagonists, there is a general lack of data regarding sex or gender differences of ADEs in the literature. In our material, gynecomastia was not one of the five most frequently reported ADEs in men (the total of five and seven reports on gynecomastia, for spironolactone and eplerenone respectively). This on the other hand could reflect the reporting system itself by the time of the study, since seriousness of the ADE could influence the propensity to submit an ADE-report to the regulatory authority.

National data from the USA show that hypertension is more frequent among women [36], and there are studies reporting different choices of antihypertensive drug classes in the treatment of women and men, with men more often receiving ACE-I and women more likely to be on diuretics [37, 38]. Gender disparities have been reported in terms of BP treatment, with women less likely to receive a beta blocker, a calcium antagonist, or an ACE-I, than a diuretic [39]. On the other hand, discontinuation of antihypertensive treatment has been found to be more common in men [40]. The findings in our study, with concomitant dispensed prescriptions of other antihypertensives being more frequent in men in general (in the different subgroups), have been reported before. In a national U.S survey among patients with treated hypertension, a lower proportion of women were taking three or more antihypertensive drugs, with men achieving BP control to a higher extent [41]. Data from the Swedish Primary Care Cardiovascular Database (SPCCD) showed that women were more often treated with diuretics and men with ACE-I although no major sex differences were seen regarding the average number of antihypertensive drug classes. BP was less well controlled in women but men interrupted their treatment to a higher extent [42]. Another study with SPCCD data showed a male predominant use of ACE-I and calcium channel blockers, not influenced by educational level, country of birth, or psychiatric disorder and therefore with sex/gender differences in side effects, suggested as a possible explanation [43].

A limitation with the design of this study is the lack of adjustment for potential confounding, as we present the linkage between ADE-reports and drug utilization at the population level rather than at the individual patient level [44]. Furthermore, the data might not represent the real incidence rate, since not all ADEs are reported. In contrast to the international ADE database of the World Health Organization (WHO), Vigibase [45], in the Swedish pharmacovigilance database SWEDIS, all reports were provided by health care professionals and causality assessed. This increases the clinical reliability compared to other pharmacovigilance studies. Vigibase also contains consumer reports, and most reports lack causality assessment. On the downside, is the lower number of reports in SWEDIS compared to Vigibase. The use of nationwide patient identity drug databases, both regarding pharmacovigilance and individualized dispensed prescription data, is an advantage of our present study [46]. To the advantage of SWEDIS is also the subclassification of ADEs into “serious” or “non-serious” when interpreting data. Findings from the literature show that women report more physical symptoms and use more medical services compared to men [47], but in theory, the propensity to report a “serious” ADE should not differ between women and men. Therefore, in this study, we aimed at reducing the possible bias of sex and gender differences in the tendency of reporting of ADEs by calculating ORs also for the “serious” ADEs as a validation of the primary analysis.

We used two methods for analyzing dispensed prescription data (DDDs/Rxs/year and DDDs/individuals/year) and they both showed the same pattern for the different subgroups of antihypertensives. For the subgroups where men have relatively higher dose exposure, the difference in the prevalence of ADE-reporting was smaller. On the other hand, in the subgroups with smaller differences in dose exposure between women and men, the higher prevalence of ADE-reporting in women was more pronounced. This indicates that dose exposure is a key factor relating to differences in ADE-reporting between women and men. An exception to this pattern were the aldosterone antagonists where we observed a higher prevalence of ADE-reports in men despite no substantial sex difference in exposure, suggesting men being more sensitive to ADEs in this case.

Persistence to drug treatment is also something to consider when analyzing sex and gender differences. A study with data from the SPCCD showed no difference between the sexes in drug class persistence between diuretics and other major antihypertensive drug classes but discontinuation was more common in men [40]. Men discontinuing their antihypertensive drug treatment could of course be a confounder to our finding of a higher prevalence of ADE-reports in women.

## Conclusion

In summary, our study on ADE-reports for antihypertensive treatment adjusted for dispensed prescription data showed a higher prevalence of ADE-reports for women in six out of ten studied antihypertensive subgroups, with a suggested linkage to dose exposure. The only subgroup with a higher prevalence of ADE-reports in men was aldosterone antagonists, and here, drug exposure was similar between the sexes.

## Electronic supplementary material

ESM 1 (DOCX 35.2 kb)
