# Supplementary material for: Sex differences in spontaneous reports on adverse drug events for common antihypertensive drugs
Source: Eur J Clin Pharmacol. 2018 May 27;74(9):1165–73. doi: 10.1007/s00228-018-2480-y (PMC6096710; doi:10.1007/s00228-018-2480-y)
Supplement: Supplementary file 1 — (DOCX 35.2 kb) [file 228_2018_2480_MOESM1_ESM.docx]

## Title:

## Sex differences in spontaneous reports on adverse drug events for common antihypertensive drugs

Journal: European Journal of Clinical Pharmacology

Authors: Diana M Rydberg, MD^1,2^, Stefan Mejyr, RN^2^, Desirée Loikas, MSc Pharm^1,4^, Karin Schenck-Gustafsson, MD, PhD^1^, Mia von Euler, MD, PhD^1-3^, Rickard E Malmström MD, PhD^1,2^

Author Affiliations: ^1^Department of Medicine Solna, Karolinska Institutet, Stockholm, Sweden; ^2^Clinical Pharmacology, Karolinska University Hospital, Stockholm, Sweden; ^3^Department of Clinical Science and Education, Södersjukhuset, Karolinska Institutet, Stockholm, Sweden; ^4^Public Healthcare Services Committee, Stockholm County Council, Stockholm, Sweden

Corresponding author’s email: [diana.rydberg@ki.se](mailto:diana.rydberg@ki.se)

Supplementary material:

**Suppl. table 1a**. Most frequently reported ADEs for ACE-Is (C09AA) 2005-2012

| **Number of ADEs (% reported for women) and numbers of these present in serious reports (% reported for women)** | | |
| --- | --- | --- |
| **Angioedema** | 265 (42) | 195 (43) |
| **Cough** | 183 (62) | 13 (69) |
| **Blood Creatinine increased** | 130 (55) | 90 (59) |
| **Hypotension** | 105 (48) | 68 (47) |
| **Hyperkalaemia** | 104 (50) | 90 (51) |

**Suppl. table 1b.** Most frequently reported ADEs for ACE-I/thiazide combinations (C09BA) 2005-2012

| **Number of ADEs (% reported for women) and numbers of these present in serious reports (% reported for women)** | | |
| --- | --- | --- |
| **Angioedema** | 24 (38) | 15 (40) |
| **Hyponatraemia** | 16 (88) | 15 (87) |
| **Cough** | 10 (60) | 0 |
| **Nausea** | 9 (78) | 3 (100) |
| **Tongue oedema** | 9 (33) | 8 (38) |

**Suppl. table 2a.** Most frequently reported ADEs for ARBs (C09CA) 2005-2012

| **Number of ADEs (% reported for women) and numbers of these present in serious reports (% reported for women)** | | |
| --- | --- | --- |
| **Hypotension** | 48 (48) | 29 (45) |
| **Angioedema** | 40 (40) | 22 (36) |
| **Blood Creatinine increased** | 34 (44) | 27 (52) |
| **Hyperkalaemia** | 27 (48) | 24 (50) |
| **Pruritus** | 24 (67) | 2 (100) |
| **Dizziness** | 24 (67) | 5 (40) |

**Suppl. table 2b.** Most frequently reported ADEs for ARB/thiazide combinations (C09DA) 2005-2012

| **Number of ADEs (% reported for women) and numbers of these present in serious reports (% reported for women)** | | |
| --- | --- | --- |
| **Hyponatraemia** | 32 (91) | 27 (93) |
| **Hypokalaemia** | 13 (92) | 9 (89) |
| **Angioedema** | 11 (18) | 3 (0) |
| **Rash** | 6 (83) | 1 (100) |
| **Blood Creatinine increased** | 6 (0) | 6 (0) |

**Suppl. table 3a.** Most frequently reported ADEs for thiazides (C03AA) 2005-2012

| **Number of ADEs (% reported for women) and numbers of these present in serious reports (% reported for women)** | | |
| --- | --- | --- |
| **Hyponatraemia** | 87 (86) | 76 (88) |
| **Hypokalaemia** | 64 (72) | 45 (82) |
| **Orthostatic hypotension** | 14 (64) | 12 (67) |
| **Hypotension** | 10 (50) | 6 (50) |
| **Nausea** | 6 (83) | 1 (100) |
| **Blood Creatinine increased** | 6 (67) | 4 (75) |

**Suppl. table 3b.** Most frequently reported ADEs for diuretics and potassium-sparing agents (C03EA) 2005-2012

| **Number of ADEs (% reported for women) and numbers of these present in serious reports (% reported for women)** | | |
| --- | --- | --- |
| **Hyponatraemia** | 132 (85) | 109 (85) |
| **Hypokalaemia** | 31 (77) | 21 (86) |
| **Hyperkalaemia** | 18 (61) | 14 (57) |
| **Hypotension** | 16 (56) | 5 (80) |
| **Orthostatic hypotension** | 12 (67) | 11 (73) |

**Suppl. table 3c.** Most frequently reported ADEs for sulfonamides (C03CA) 2005-2012

| **Number of ADEs (% reported for women) and numbers of these present in serious reports (% reported for women)** | | |
| --- | --- | --- |
| **Hypotension** | 58 (40) | 36 (47) |
| **Hyponatraemia** | 46 (80) | 37 (81) |
| **Orthostatic hypotension** | 42 (45) | 37 (43) |
| **Hypokalaemia** | 38 (79) | 27 (81) |
| **Dehydration** | 24 (50) | 22 (55) |
| **Blood Creatinine increased** | 24 (63) | 20 (60) |

**Suppl. table 3d.** Most frequently reported ADEs for aldosterone antagonists (C03DA) 2005-2012

| **Number of ADEs (% reported for women) and numbers of these present in serious reports (% reported for women)** | | |
| --- | --- | --- |
| **Hyperkalaemia** | 114 (43) | 84 (43) |
| **Blood Creatinine increased** | 59 (49) | 48 (46) |
| **Hypotension** | 25 (44) | 23 (48) |
| **Hyponatraemia** | 24 (75) | 17 (71) |
| **Renal failure** | 15 (67) | 14 (71) |

**Suppl. table 4.** Most frequently reported ADEs for dihydropyridines (C08CA) 2005-2012

| **Number of ADEs (% reported for women) and numbers of these present in serious reports (% reported for women)** | | |
| --- | --- | --- |
| **Oedema legs** | 72 (68) | 13 (62) |
| **Hypotension** | 58 (55) | 30 (57) |
| **Orthostatic hypotension** | 46 (61) | 36 (61) |
| **Pruritus** | 32 (59) | 3 (33) |
| **Dizziness** | 31 (58) | 11 (36) |
|  |  |  |

**Suppl. table 5.** Most frequently reported ADEs for selective beta blockers (C07AB) 2005-2012

| **Number of ADEs (% reported for women) and numbers of these present in serious reports (% reported for women)** | | |
| --- | --- | --- |
| **Bradycardia** | 355 (46) | 264 (47) |
| **Hypotension** | 113 (48) | 68 (47) |
| **Orthostatic hypotension** | 70 (49) | 58 (50) |
| **Syncope** | 51 (43) | 50 (44) |
| **Dizziness** | 45 (58) | 23 (52) |
|  |  |  |

**Suppl. table 6a**. Total reports of ACE-Is (C09AA) and most commonly suspected co-medication

|  | Total reports  women |  | Total reports men |
| --- | --- | --- | --- |
| ACE-Is | 666 | ACE-Is | 625 |
| **Co-medication** | | | |
| Aldostrone antagonists | 44 | Selective beta blockers | 42 |
| Sulfonamides | 41 | Aldosterone antagonists | 34 |
| Selective beta blockers | 36 | Sulfonamides | 27 |

**Suppl. table 6b**. Total reports of ACE-I/thiazide combinations (C09BA) and most commonly suspected co-medication

|  | Total reports  women |  | Total reports men |
| --- | --- | --- | --- |
| ACE-I/ thiazide combinations | 57 | ACE-I/thiazide combinations | 44 |
| **Co-medication** | | | |
| Selective beta blockers | 5 | Selective beta blockers | 4 |
| Sulfonamides | 4 | Dihydropyridines | 4 |
| Aldosterone antagonists, proton pump inhibitors | 3 | ACE-Is | 4 |

**Suppl. table 7a.** Total reports of ARBs (C09CA) and most commonly suspected co-medication

|  | Total reports  women |  | Total reports men |
| --- | --- | --- | --- |
| ARBs | 248 | ARBs | 199 |
| **Co-medication** | | | |
| Aldosterone antagonists | 18 | Sulfonamides | 17 |
| Sulfonamides | 15 | Selective beta blockers | 17 |
| Thiazides | 12 | Aldosterone antagonists | 14 |

**Suppl. table 7b.** Total reports of ARB/thiazide combinations (C09DA) and most commonly suspected co-medication

|  | Total reports  women |  | Total reports men |
| --- | --- | --- | --- |
| ARB/thiazide combinations | 88 | ARB/thiazide combinations | 42 |
| **Co-medication** | | | |
| Sulfonamides | 7 | Acetic acid derivates (M01AB) | 3 |
| Selective beta blockers | 6 | Biguanides, PAI (platelet aggr inhib), | 2 |
| SSRIs | 5 | Opium alkaloids, sulfonamides | 2 |

**Suppl. table 8a**. Total reports of thiazides (C03AA) and most commonly suspected co-medication

|  | Total reports women |  | Total reports men |
| --- | --- | --- | --- |
| Thiazides | 160 | Thiazides | 62 |
| **Co-medication** | | | |
| ACE-Is | 15 | ACE-Is | 8 |
| SSRIs | 13 | Dihydropyridines | 3 |
| ARBs | 12 | Sulfonamides | 2 |

**Suppl. table 8b.** Total reports of diuretics and potassium-sparing agents (C03EA) and most commonly suspected co-medication

|  | Total reports  women |  | Total reports men |
| --- | --- | --- | --- |
| Diuretics and potassium-sparing agents | 196 | Diuretics and potassium-sparing agents | 60 |
| **Co-medication** | | | |
| SSRIs | 23 | Sulfonamides | 5 |
| ACE-Is | 15 | Selective beta blockers | 5 |
| Sulfonamides | 10 | ACE-Is, ARBs | 4 |

**Suppl. table 8c**. Total reports of sulfonamides (C03CA) and most commonly suspected co-medication

|  | Total reports  women |  | Total reports men |
| --- | --- | --- | --- |
| Sulfonamides | 177 | Sulfonamides | 130 |
| **Co-medication** | | | |
| ACE-Is | 41 | ACE-Is | 28 |
| Aldosterone antagonists | 29 | Selective beta blockers | 24 |
| Selective beta blockers | 19 | ARBs | 18 |

**Suppl. table 8d**. Total reports of aldosterone antagonists (C03DA) and most commonly suspected co-medication

|  | Total reports  women |  | Total reports men |
| --- | --- | --- | --- |
| Aldosterone antagonists | 125 | Aldosterone antagonists | 121 |
| **Co-medication** | | | |
| ACE-Is | 44 | ACE-Is | 33 |
| Sulfonamides | 29 | ARBs | 14 |
| ARBs | 18 | Sulfonamides, selective beta blockers | 13 |

**Suppl.table 9**. Total reports of dihydropyridines (C08CA) and most commonly suspected co-medication

|  | Total reports  women |  | Total reports men |
| --- | --- | --- | --- |
| Dihydropyridines | 293 | Dihydropyridines | 204 |
| **Co-medication** | | | |
| ACE-Is | 23 | Selective beta blockers | 17 |
| Selective beta blockers | 21 | ACE-Is | 16 |
| ARBs | 12 | Sulfonamides | 10 |

**Suppl. table 10.** Total reports of selective beta blockers (C07AB) and most commonly suspected co-medication

|  | Total reports  women |  | Total reports men |
| --- | --- | --- | --- |
| Selective beta blockers | 436 | Selective beta blockers | 435 |
| **Co-medication** | | | |
| Digitalis glycosides | 51 | ACE-Is | 42 |
| ACE-Is | 36 | Digitalis glycosides | 29 |
| Dihydropyridines | 21 | Sulfonamides | 24 |

**Suppl. table 11.** Number of patients that, at least once during July 2005-December 2012, dispensed **ACE-Is** and the proportion of these patients, with the four most commonly co-prescribed drugs for antihypertensive treatment, the same day (six months data is also shown).

|  | ACE-Is | ACE-Is and selective beta blockers | | ACE-Is and sulfonamides | | ACE-Is and dihydropyridines | | ACE-Is and thiazides | |
| --- | --- | --- | --- | --- | --- | --- | --- | --- | --- |
|  |  | Same  day | Six  months | Same  day | Six  months | Same  day | Six  months | Same  day | Six  months |
| Total Rx | 1 144 397 | 448 160  (39%) | 575 021  (50%) | 257 701  (23%) | 328 472  (29%) | 247 718  (22%) | 400 291  (35%) | 128 291  (11%) | 197 049  (17%) |
| Women | 534 883 | 195 711  (37%) | 266 423  (50%) | 128 518  (24%) | 166 894  (32%) | 103 577  (19%) | 185 352  (35%) | 64 680  (12%) | 105 839  (20%) |
| Men | 609 514 | 252 449  (41%) | 308 598  (51%) | 129 183  (21%) | 161 578  (27%) | 144 141  (24%) | 214 939  (35%) | 63 611  (10%) | 91 210  (15%) |
| OR^1^ | NA | 0.88 | 0.98 | 1.13 | 1.18 | 0.82 | 0.98 | 1.16 | 1.32 |
| CI^2^ | NA | 0.88-0.89 | 0.98-0.99 | 1.12-1.14 | 1.17-1.19 | 0.81-0.83 | 0.98-0.99 | 1.15-1.17 | 1.31-1.34 |

^1^Women vs men

^2^0.95 Confidence Interval of the ORs

**Suppl. table 12**. Number of patients that, at least once during July 2005-December 2012, dispensed **ARBs** and the proportion of these patients, with the four most commonly co-prescribed drugs for antihypertensive treatment, the same day (six months data is also shown).

|  | ARBs | ARBs and selective beta blockers | | ARBs and dihydropyridines | | ARBs and sulfonamides | | ARBs and thiazides | |
| --- | --- | --- | --- | --- | --- | --- | --- | --- | --- |
|  |  | Same  day | Six  months | Same  day | Six  months | Same  day | Six  months | Same  day | Six  Months |
| Total Rx | 599 725 | 221 589  (37%) | 298 212  (50%) | 148 122  (25%) | 236 827  (39%) | 126 830  (21%) | 174 106  (29%) | 74 214  (12%) | 113 358  (19%) |
| Women | 308 433 | 113 324  (37%) | 155 686  (50%) | 70 067  (23%) | 119 965  (39%) | 70 380  (23%) | 96 922  (31%) | 42 678  (14%) | 66 419  (22%) |
| Men | 291 292 | 108 265  (37%) | 142 526  (49%) | 78 055  (27%) | 116 862  (40%) | 56 450  (19%) | 77 184  (26%) | 31 536  (11%) | 46 939  (16%) |
| OR^1^ | NA | 0.99 | 1.03 | 0.85 | 0.97 | 1.18 | 1.19 | 1.28 | 1.34 |
| CI^2^ | NA | 0.98-1.00 | 1.02-1.04 | 0.84-0.86 | 0.96-0.98 | 1.16-1.19 | 1.17-1.20 | 1.26-1.30 | 1.32-1.35 |

^1^Women vs men

^2^0.95 Confidence Interval of the ORs

**Suppl.table 13a**. Number of patients that, at least once during July 2005-December 2012, dispensed **thiazides** and the proportion of these patients, with the four most commonly prescribed drugs for antihypertensive treatment, the same day (six months data is also shown).

|  | Thiazides | Thiazides and selective beta blockers | | Thiazides and ACE-Is | | Thiazides and dihydropyridines | | Thiazides and ARBs | |
| --- | --- | --- | --- | --- | --- | --- | --- | --- | --- |
|  |  | Same  day | Six  months | Same  day | Six  months | Same  day | Six  months | Same  day | Six  Months |
| Total Rx | 419 343 | 149 099  (36%) | 204 155  (49%) | 128 291  (31%) | 197 049  (47%) | 104 535  (25%) | 167 695  (40%) | 74 214  (18%) | 113 358  (27%) |
| Women | 248 051 | 88 189  (36%) | 121 142  (49%) | 64 680  (26%) | 105 839  (43%) | 55 233  (22%) | 92 851  (37%) | 42 678  (17%) | 66 419  (27%) |
| Men | 171 292 | 60 910  (36%) | 83 013  (48%) | 63 611  (37%) | 91 210  (53%) | 49 302  (29%) | 74 844  (44%) | 31 536  (18%) | 46 939  (27%) |
| OR^1^ | NA | 1.00 | 1.01 | 0.70 | 0.80 | 0.77 | 0.86 | 0.93 | 0.98 |
| CI^2^ | NA | 0.99-1.01 | 1.00-1.02 | 0.69-0.71 | 0.79-0.81 | 0.76-0.78 | 0.85-0.87 | 0.92-0.95 | 0.96-0.99 |

**Suppl.table 13b**. Number of patients that, at least once during July 2005-December 2012, dispensed **diuretics and potassium-sparing agents (C03EA)** and the proportion of these patients, with the four most commonly prescribed drugs for antihypertensive treatment, the same day (six months data is also shown).

|  | Diuretics and potassium sparing agents | C03EA and dihydropyridines | | C03EA and ACE-Is | | C03EA and ARBs | | C03EA and selective beta blockers | |
| --- | --- | --- | --- | --- | --- | --- | --- | --- | --- |
|  |  | Same  day | Six  months | Same  day | Six  months | Same  day | Six  months | Same  day | Six  Months |
| Total Rx | 244 373 | 43 838  (18%) | 76 150  (31%) | 37 492  (15%) | 81 579  (33%) | 22 720  (9%) | 44 214  (18%) | 21 677  (9%) | 111 546  (46%) |
| Women | 163 231 | 26 613  (16%) | 48 150  (29%) | 21 078  (13%) | 50 244  (31%) | 14 238  (9%) | 28 928  (18%) | 14 332  (9%) | 74 159  (45%) |
| Men | 81 142 | 17 225  (21%) | 28 000  (35%) | 16 414  (20%) | 31 335  (39%) | 8 482  (10%) | 15 286  (19%) | 7 345  (9%) | 37 387  (46%) |
| OR^1^ | NA | 0.77 | 0.85 | 0.64 | 0.80 | 0.83 | 0.94 | 0.97 | 0.99 |
| CI^2^ | NA | 0.75-0.78 | 0.84-0.87 | 0.62-0.65 | 0.78-0.81 | 0.81-0.86 | 0.92-0.96 | 0.94-1.00 | 0.97-1.00 |

**Suppl.table 13c.** Number of patients that, at least once during July 2005-December 2012, dispensed **sulfonamides** and the proportion of these patients, with the three most commonly prescribed drugs for antihypertensive treatment, the same day (six months data is also shown).

|  | Sulfonamides | Sulfonamides and selective beta blockers | | Sulfonamides and ACE-Is | | Sulfonamides and dihydropyridines | | Sulfonamides and ARBs | |
| --- | --- | --- | --- | --- | --- | --- | --- | --- | --- |
|  |  | Same  day | Six  months | Same  day | Six  months | Same  day | Six  months | Same  day | Six  Months |
| Total Rx | 857 345 | 371 105  (43%) | 456 264  (53%) | 257 701  (30%) | 328 472  (38%) | 171 050  (20%) | 253 133  (30%) | 126 830  (15%) | 174 106  (20%) |
| Women | 501 956 | 202 642  (40%) | 249 638  (50%) | 128 518  (26%) | 166 894  (33%) | 93 747  (19%) | 139 340  (28%) | 70 380  (14%) | 96 922  (19%) |
| Men | 355 389 | 168 463  (47%) | 206 626  (58%) | 129 183  (36%) | 161 578  (45%) | 77 303  (22%) | 113 793  (32%) | 56 450  (16%) | 77 184  (22%) |
| OR^1^ | NA | 0.85 | 0.86 | 0.70 | 0.73 | 0.86 | 0.87 | 0.88 | 0.89 |
| CI^2^ | NA | 0.85-0.86 | 0.85-0.86 | 0.70-0.71 | 0.73-0.74 | 0.85-0.87 | 0.86-0.87 | 0.87-0.89 | 0.88-0.90 |

**Suppl. table 13d**. Number of patients that, at least once during July 2005-December 2012, dispensed **aldosterone antagonists** and the proportion of these patients, with the two most commonly prescribed drugs for antihypertensive treatment, the same day (six months data is also shown).

|  | Aldosterone antagonists | Aldosterone antagonists and selective beta blockers | | Aldosterone antagonists and ACE-Is | | Aldosterone antagonists and dihydropyridines | | Aldosterone antagonists and ARBs | |
| --- | --- | --- | --- | --- | --- | --- | --- | --- | --- |
|  |  | Same  day | Six  months | Same  day | Six  months | Same  day | Six  months | Same  day | Six  Months |
| Total Rx | 258 422 | 117 945  (46%) | 151 129  (58%) | 71 180  (28%) | 101 972  (39%) | 40 421  (16%) | 68 802  (27%) | 36 933  (14%) | 57 311  (22%) |
| Women | 149 418 | 63 712  (43%) | 81 203  (54%) | 32 482  (22%) | 48 941  (33%) | 22 223  (15%) | 38 055  (25%) | 19 462  (13%) | 30 590  (20%) |
| Men | 109 004 | 54 233  (50%) | 69 926  (64%) | 38 698  (36%) | 53 031  (49%) | 18 198  (17%) | 30 747  (28%) | 17 471  (16%) | 26 721  (25%) |
| OR^1^ | NA | 0.86 | 0.85 | 0.61 | 0.67 | 0.89 | 0.90 | 0.81 | 0.84 |
| CI^2^ | NA | 0.85-0.87 | 0.84-0.86 | 0.60-0.62 | 0.66-0.68 | 0.87-0.91 | 0.89-0.92 | 0.80-0.83 | 0.82-0.85 |

^1^Women vs men

^2^0.95 Confidence Interval of the ORs

**Suppl. table 14**. Number of patients that, at least once during July 2005-December 2012, dispensed **dihydropyridines** and the proportion of these patients, with the four most commonly prescribed drugs for antihypertensive treatment, the same day (six months data is also shown).

|  | Dihydro-  pyridines | Dihydropyridines and selective beta blockers | | Dihydropyridines  and ACE-Is | | Dihydropyridines and sulfonamides | | Dihydropyridines and ARBs | |
| --- | --- | --- | --- | --- | --- | --- | --- | --- | --- |
|  |  | Same  day | Six  months | Same  day | Six  months | Same  day | Six  months | Same  day | Six  months |
| Total Rx | 918 184 | 395 150  (43%) | 502 419  (55%) | 247 718  (27%) | 400 291  (44%) | 171 050  (19%) | 253 133  (28%) | 148 122  (16%) | 236 827  (26%) |
| Women | 464 926 | 196 490  (42%) | 256 522  (55%) | 103 577  (22%) | 185 352  (40%) | 93 747  (20%) | 139 340  (30%) | 70 067  (15%) | 119 965  (26%) |
| Men | 453 258 | 198 660  (44%) | 245 897  (54%) | 144 141  (32%) | 214 939  (47%) | 77 303  (17%) | 113 793  (25%) | 78 055  (17%) | 116 862  (26%) |
| OR^1^ | NA | 0.96 | 1.02 | 0.70 | 0.84 | 1.18 | 1.19 | 0.88 | 1.00 |
| CI^2^ | NA | 0.96-0.97 | 1.01-1.02 | 0.69-0.71 | 0.83-0.85 | 1.17-1.19 | 1.18-1.20 | 0.87-0.88 | 0.99-1.01 |

^1^Women vs men

^2^0.95 Confidence Interval of the ORs

**Suppl.table 15.** Number of patients that, at least once during July 2005-December 2012, dispensed **selective beta blockers** and the proportion of these patients, with the four most commonly prescribed drugs for antihypertensive treatment, the same day (six months data is also shown).

|  | Selective beta blockers | Selective beta blockers and ACE-Is | | Selective beta blockers and dihydropyridines | | Selective beta blockers and sulfonamides | | Selective beta blockers and ARBs | |
| --- | --- | --- | --- | --- | --- | --- | --- | --- | --- |
|  |  | Same  day | Six  months | Same  day | Six  months | Same  day | Six  months | Same  day | Six  months |
| Total Rx | 1 392 619 | 448 160  (32%) | 575 021  (41%) | 395 150  (28%) | 502 419  (36%) | 371 105  (27%) | 456 264  (33%) | 221 589  (16%) | 298 212  (21%) |
| Women | 726 291 | 195 711  (27%) | 266 423  (37%) | 196 490  (27%) | 256 522  (35%) | 202 642  (28%) | 249 638  (34%) | 113 324  (16%) | 155 686  (21%) |
| Men | 666 328 | 252 449  (38%) | 308 598  (46%) | 198 660  (30%) | 245 897  (37%) | 168 463  (25%) | 206 626  (31%) | 108 265  (16%) | 142 526  (21%) |
| OR^1^ | NA | 0.71 | 0.79 | 0.91 | 0.96 | 1.10 | 1.11 | 0.96 | 1.00 |
| CI^2^ | NA | 0.71-0.72 | 0.79-0.80 | 0.90-0.91 | 0.95-0.96 | 1.10-1.11 | 1.10-1.12 | 0.95-0.97 | 0.99-1.01 |

^1^Women vs men

^2^0.95 Confidence Interval of the ORs
